# A protocol of systematic review and meta-analysis of narrow band imaging endoscopy in detection of early gastric cancer

**DOI:** 10.1097/MD.0000000000021420

**Published:** 2020-08-14

**Authors:** Li Zhang, Xiao-yu Liu, Gang Zhong, Zhi Xin, Xiang-Yu Sun, Zhen-Yu Wang

**Affiliations:** a Endoscopy Center, Tianjin Nankai Hospital, Tianjin; b Oncology Department, Yulin Second Hospital, Xianan, Shanxi Province, China.

**Keywords:** early detection, early gastric cancer, meta-analysis, mortality

## Abstract

**Background:**

Early gastric cancer is the fifth common malignancy and the third leading cause of cancer death throughout the world. However, it is not clear how endoscopic screening for early gastric cancer affects incidence or mortality. We performed a systematic review and meta-analysis to evaluate the relationship between endoscopic screening for the mortality and incidence of early gastric cancer.

**Materials and methods:**

This protocol established in this study has been reported following the Preferred Reporting Items for Systematic Review and Meta-Analysis Protocols. Web of Science, PubMed, EMBASE, and the Cochrane Library were searched for cohort and case-control studies in cases without early gastric cancer until March 31, 2020. We will use a combination of Medical Subject Heading and free-text terms with various synonyms to search based on the Eligibility criteria. Two investigators independently reviewed the included studies and extracted relevant data. The relative risk and 95% confidence intervals were used as effect estimate. I-square test, substantial heterogeneity, sensitivity analysis, and publication bias assessment will be performed accordingly. Stata 15.0 and Review Manger 5.3 are used for meta-analysis and systematic review.

**Results:**

The results will be published in a peer-reviewed journal.

**Conclusion:**

The results of this review will be widely disseminated through peer-reviewed publications and conference presentations. This evidence may also provide helpful evidence of whether endoscopic screening would reduce the mortality and incidence of early gastric cancer.

**PROSPERO registration number:**

CRD42020171053

## 1 Introduction

Early gastric cancer is the fifth common malignancy and the third leading cause of cancer death throughout the world, with 952,600 new cases and 723,100 deaths in 2012.^[1]^ The 5-year survival rate was 90% for postresection early-stage early gastric cancer patients, while it was nearly 10% for advanced-stage patients.^[2]^ Although surgery is the standard treatment for early gastric cancer, early detection, diagnosis, and treatment is the only way to reduce mortality.^[3]^ Therefore, it is significant to have early detection and early treatment.

Most studies have shown a 40% to 60% decrease in the mortality of early gastric cancer in those who have been screened using photofluorography, though data are inconsistent.^[4,5]^ For patients with positive findings using photofluorography, further examination with endoscopy is recommended.^[6]^ One study found that the finding ratio of endoscopic examination was approximately 4.6 times higher than photofluorography in detecting early gastric cancer.^[7]^ However, it is not clear how endoscopic screening for early gastric cancer affects incidence or mortality.

Endoscopy was introduced to the national screening program with an upper gastrointestinal series (UGIS).^[8]^ Participants can choose either endoscopy or UGIS depending on their individual preference. Similarly, screening using endoscopy was more closely associated with a diagnosis of localized early gastric cancer than screening using UGIS.^[9]^ Although benefits have been obtained in detection, diagnosis, and treatment since introducing endoscopy in the management of early gastric cancer, the effects of endoscopy as a screening tool on early gastric cancer mortality and incidence are controversial.^[10]^ Given that there is a lack of enough clinical data,^[11]^ we conducted this systematic review of case-control and cohort studies, to evaluate the relationship between endoscopic screening and early gastric cancer mortality and incidence.

## 2 Study aim

The aim of our study is to provide helpful evidence of whether endoscopic screening would reduce the mortality and incidence of early gastric cancer. A better understanding of early gastric cancer guides the use of endoscopic screening.

## 3 Methods

The protocol of our MAs followed the guideline of the Preferred Reporting Items for Systematic Review and Meta-Analysis Protocols (PRISMA-P) recommendations.^[12]^ It has been registered with International Prospective Register of Systematic Reviews (PROSPERO) as CRD42020171053 (https://www.crd.york.ac.uk/prospero/display_record.php?ID=CRD42020171053).

### 3.1 Eligibility criteria

#### 3.1.1 Types of studies

Prospective cohort, retrospective cohort, or case-control studies of endoscopic screening to forecast the mortality and incidence of early gastric cancer will be included to pool and review in this study. Studies assessing the effect of endoscopic screening only in individuals with premalignant conditions,^[13]^ Barrett esophagus,^[14]^ and *Helicobacter pylori*^[15]^ (*H pylori*) infection were excluded.

#### 3.1.2 Types of participants and interventions

Studies included adults aged 18 years old and older without diagnosis of early gastric cancer in the general population. Intervention must be at least once of endoscopic screening including mass screening, opportunistic screening, followed by entry into surveillance or not.

#### 3.1.3 Types of outcome

Outcomes will include mortality or incidence (including studies with a combine outcome of esophago-early gastric cancer mortality or incidence) confirmed by pathological diagnosis, International Classification of Diseases codes, or records from government registration, presented as risk ratio, odds ratio, hazard ratios, standardized incidence ratio, standardized mortality ratio, and associated 95% confidence intervals (CIs).

### 3.2 Search strategy

Web of Science, PubMed, EMBASE, and the Cochrane Library were searched for cohort and case-control studies in cases without early gastric cancer until March 31, 2020. The MeSH search and text word will be used with the terms related to early gastric cancer and endoscopic screening. To perform a comprehensive and focused search, experienced systematic review researchers will be invited to develop a search strategy. The plan searched terms are as follows: early gastric cancers, stomach neoplasms, gastric carcinomas, endoscopic screening, etc. An example of search strategy for PubMed database shown in Table 1 will be modified and used for the other databases. The reference lists of all relevant studies will be searched for additional relevant studies not retrieved from the electronic database search.

**Table 1:**
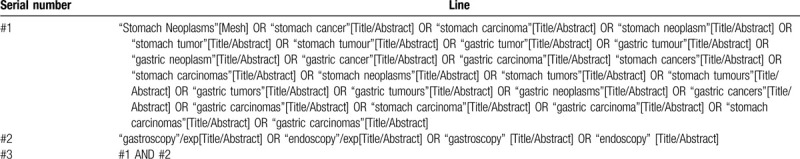
Searching strategy in PubMed.

### 3.3 Study selection

**Figure 1:**
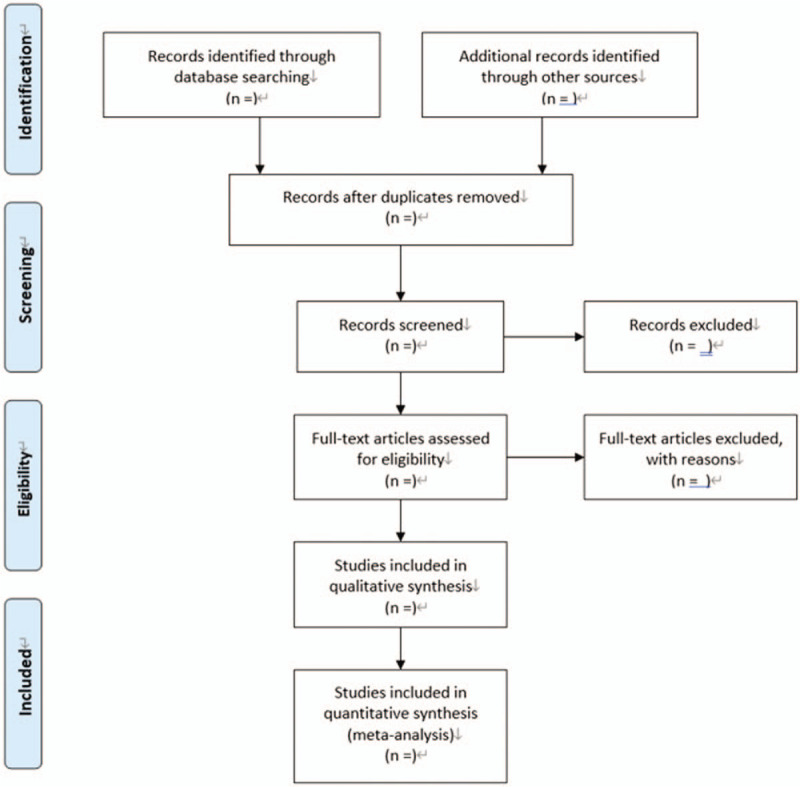
Flow diagram: selection process for the studies.

All initial records from 4 electronic databases will be imported into the web-based systematic review Rayyan software.^[16]^ First, the titles and abstracts of records will be reviewed independently by 2 reviewers to identify potential trials according to eligibility criteria. Then, full text of all potentially relevant trials will be downloaded to make sure eligible trials. Any conflict will be resolved by discussion. A flow diagram (Fig. 1) will be used to describe the selection process of eligible papers.

### 3.4 Data extraction and management

The data will be extracted out by 2 independent reviewers in accordance with the Cochrane Handbook of Systematic Reviews of Interventions. Two investigators will independently screen all the included studies to extract the following data: name of the first author, publication year, study design, country, intervention, comparator, study period, sample size, numbers of outcomes, frequency of endoscopic screening, timing of endoscopic screening, age at enrollment, sex, duration of follow-up, adjustments, and effect estimates.

### 3.5 Risk of bias of individual study and quality assessment

Two reviewers will evaluate independently the risk of bias of included studies using a modified version of Cochrane tool^[17]^ in which we will check for allocation concealment, blinding, incomplete outcome data, selective reporting, and other bias, each of which makes high-risk, low-risk, and unclear grades. The Newcastle-Ottawa Quality Assessment Scale^[18]^ was employed to assess the quality of each of the included studies. Any discrepancy was resolved by discussion or by a third reviewer.

### 3.6 Data analyses

The effect estimate of interest will be the relative risk. Statistical analyses will be performed using Review Manager 5.3 statistical software and Stata 15.0 software. The outcomes will be presented as the relative risk, mean difference, or standardized mean difference and its 95% CI. The statistical significance will be assessed for *P*<.05, and moderate to high levels of heterogeneity will be considered for I^2^ > 50%.^[19]^ A fixed effects model will be used if there is no statistical heterogeneity across the studies; otherwise, the random effects model will be considered.

### 3.7 Publication bias

If included studies were more than 10, funnel plot will be used to identify the possible publication bias. Additionally, Egg regression and Begg tests will be utilized to detect the funnel plot asymmetry.^[20]^

### 3.8 Subgroup analysis

If there is enough research, we will conduct a subgroup analysis to investigate differences in age, gender, etc.

## 4 Discussion

It is not clear how endoscopic screening for early gastric cancer affects incidence or mortality. This systematic review and meta-analysis will evaluate the relationship between endoscopic screening for the mortality and incidence of early gastric cancer. The results of this review will be widely disseminated through peer-reviewed publications and conference presentations. This evidence may also provide helpful evidence of whether endoscopic screening would reduce the mortality and incidence of early gastric cancer.

## Author contributions

XXXX.
